# Dysphagia is a strong predictor of death and functional dependence at three months post-stroke

**DOI:** 10.1590/0004-282X-ANP-2021-0127

**Published:** 2022-02-21

**Authors:** Aline Cristina PACHECO-CASTILHO, Rubia Poliana Crisóstomo MIRANDA, Ana Maria Queirós NORBERTO, Diandra Bosi FAVORETTO, Brunna Pileggi RIMOLI, Luciana Bezerra de Mello ALVES, Karina Tavares WEBER, Taiza Elaine Grespan SANTOS, Julio Cesar MORIGUTI, João Pereira LEITE, Roberto Oliveira DANTAS, Rosemary MARTINO, Octávio Marques PONTES-NETO

**Affiliations:** 1 Universidade de São Paulo, Faculdade de Medicina de Ribeirão Preto, Departamento de Neurociências e Ciências do Comportamento, Ribeirão Preto SP, Brazil.; 2 Universidade de São Paulo, Faculdade de Medicina de Ribeirão Preto, Departamento de Oftalmologia, Otorrinolaringologia e Cirurgia de Cabeça e Pescoço, Ribeirão Preto SP, Brazil.; 3 Universidade de São Paulo, Faculdade de Medicina de Ribeirão Preto, Departamento de Clínica Médica, Ribeirão Preto SP, Brazil.; 4 University of Toronto, Graduate Department of Rehabilitation Science, Department of Speech-Language Pathology, Toronto, Ontario, Canada.

**Keywords:** Deglutition, Deglutition Disorders, Stroke, Outcome Assessment, Health Care, Deglutição, Transtornos de Deglutição, Acidente Vascular Cerebral, Avaliação de Resultados em Cuidados de Saúde

## Abstract

**Background:**

Few Brazilian studies investigated risk factors for dysphagia and associated complications in a large cohort.

**Objective:**

To investigate frequency, predictors, and associated outcomes of dysphagia in patients up to three months post-stroke.

**Methods:**

Prospective cohort study of consecutively admitted patients in a specialized center for acute stroke. Patients with a transient ischemic attack, subarachnoid hemorrhage, cerebral venous thrombosis, hemorrhagic stroke with secondary cause, non-acute stroke, or those who did not consent to participate were excluded. Swallowing was evaluated by speech language pathologists using Volume-Viscosity Swallow Test. General function at three months post-stroke was assessed using the following instruments: Modified Rankin scale, Barthel Index and Functional Independence Measure.

**Results:**

A total of 831 patients were admitted and 305 patients were included according to the inclusion and exclusion criteria. The mean age of patients was 63.6±13.3 years, mean time from stroke to swallowing assessment was 4.2±4.1 days, and 45.2% of the patients had dysphagia. Age (OR=1.02; 95%CI 1.00-1.04; p=0.017), known medical history of obstructive sleep apnea (OR=5.13; 95%CI 1.74-15.15; p=0.003), and stroke severity at hospital admission (OR=1.10; 95%CI 1.06-1.15; p<0.001) were independently associated with dysphagia. Dysphagia (OR=3.78; 95%CI 2.16-6.61; p<0.001) and stroke severity (OR=1.05; 95%CI 1.00-1.09; p=0.024) were independently associated with death or functional dependence at three months.

**Conclusions:**

Dysphagia was present in almost half of stroke patients. Age, obstructive sleep apnea, and stroke severity were predictors of dysphagia, which was independently associated with death or functional dependence at three months.

## RESUMO

**Antecedentes:**

Poucos estudos brasileiros investigaram fatores de risco para disfagia e suas complicações associadas em uma grande coorte.

**Objetivo:**

Investigar frequência, preditores e desfechos associados da disfagia em pacientes até três meses após acidente vascular cerebral (AVC).

**Métodos:**

Selecionamos pacientes admitidos consecutivamente em um centro especializado em AVC agudo. Excluímos pacientes com ataque isquêmico transitório, hemorragia subaracnóidea, trombose venosa cerebral, AVC hemorrágico de causa secundária, AVC não agudo ou aqueles que não consentiram em participar. A deglutição foi avaliada por fonoaudiólogos, por meio do teste de deglutição de volume-viscosidade. A função geral foi avaliada usando-se escala de Rankin modificada, índice de Barthel e medida de independência funcional.

**Resultados:**

Foram admitidos 831 pacientes e incluídos 305. A idade média foi 63,6±13,3 anos, o tempo médio da avaliação foi 4,2±4,1 dias e 45,2% apresentavam disfagia. Idade (razão de chances [OR] 1,02; intervalo de confiança [IC95%] 1,00-1,04; p=0,017), história médica conhecida de apneia obstrutiva do sono (OR=5,13; IC95% 1,74-15,15; p=0,003) e gravidade do AVC na admissão hospitalar (OR=1,10; IC95% 1,06-1,15; p<0,001) foram independentemente associados à disfagia. Disfagia (OR=3,78; IC95% 2,16-6,61; p<0,001) e gravidade do AVC (OR=1,05; IC95% 1,00-1,09; p=0,024) foram independentemente associadas com morte ou dependência funcional em três meses.

**Conclusões:**

A disfagia esteve presente em quase metade dos pacientes com AVC. Idade, apneia obstrutiva do sono e gravidade do AVC foram preditores de disfagia, que esteve independentemente associada com morte ou dependência funcional em três meses.

## INTRODUCTION

Dysphagia is common in post-stroke individuals^1^ and contributes to worse long-term outcomes, including functional dependence^2^ ^,^ ^3^ ^,^ ^4^ ^,^ ^5^, institutionalization^2^ ^,^ ^4^ ^,^ ^5^ ^,^ ^6^ ^,^ ^7^ and increased mortality^2^ ^,^ ^3^ ^,^ ^4^ ^,^ ^5^ ^,^ ^6^ ^,^ ^7^ ^,^ ^8^ ^,^ ^9^. Some studies with stroke individuals have demonstrated that the presence of dysphagia is associated with age^2^ ^,^ ^5^ ^,^ ^6^ ^,^ ^7^ ^,^ ^8^ ^,^ ^10^, female sex^2^ ^,^ ^5^ ^,^ ^6^, stroke severity^2^ ^,^ ^4^ ^,^ ^5^ ^,^ ^7^ ^,^ ^8^ ^,^ ^11^, hemorrhagic stroke^6^ ^,^ ^8^, lesion in the left hemisphere^9^, stroke involving total anterior circulation^6^, stroke with involvement of the middle cerebral artery^8^, brain stem lesion^10^, prior stroke^5^ ^,^ ^9^ ^,^ ^12^, hypertension^2^ ^,^ ^5^, diabetes^10^ and atrial fibrillation^2^ ^,^ ^6^ ^,^ ^8^. However, the factors associated with dysphagia in stroke individuals are not well established^5^ ^,^ ^12^.

In Brazil, the frequency of dysphagia in individuals with stroke is high compared to developed countries^13^. Nevertheless, only two Brazilian studies were conducted prospectively with a large sample of stroke individuals to identify the factors associated with dysphagia and the impact of dysphagia on this population^9^ ^,^ ^14^. In addition, these studies have explored few risk factors and did not report blinded assessments of outcomes.

Knowledge about factors associated with dysphagia and the impact of dysphagia on outcomes is important for stroke teams because it can provide information on what to expect in the assessment and prognosis of these individuals, and therefore may contribute to the planning of early preventive measures.

Thus, the aims of this study were to investigate the factors associated with dysphagia and to assess the impact of dysphagia on sub-acute clinical outcomes in stroke individuals in a large cohort prospectively and with blinded assessments of outcomes.

## METHODS

### Design of the study

To investigate the frequency of dysphagia and its associated factors, we performed a cross-sectional study, and to assess the impact of dysphagia on outcomes, we conducted a cohort study.

### Subjects

All consecutive eligible individuals admitted to the emergency unit of a tertiary academic Brazilian hospital were approached and gave consent. Eligible individuals were those that met the following criteria: age ≥18 years and had a medical diagnosis of any stroke event confirmed by CT scan and/or MRI findings. Individuals with a transient ischemic attack, subarachnoid hemorrhage, cerebral venous thrombosis, hemorrhagic stroke with secondary cause, non-acute stroke (>10 days after last seen normal), or those who did not consent were excluded. This study was approved by the Ethics Committee of our institution. Individuals that were discharged from hospital before swallowing assessment or those that were not able to be assessed due to clinical conditions were also excluded.

### Data source

#### Demographic and clinical information

All individuals were initially screened by research coordinators as part of the admitting process with an institution-specific stroke registry. The data were collected prospectively as per standard of care and included age, sex, premorbid functional status, potential risk factors for stroke, admission/discharge dates, stroke details, tube feeding, overall function, and in-hospital death.

#### Stroke characteristics

The neuroimaging analysis was performed by neurovascular neurologists blinded to dysphagia diagnosis. The software used for analysis was Weasis v2.03. Exams were classified according to stroke type and hemisphere injured. Ischemic strokes were classified into “lacunar”, “cerebellar”, “cortical” and “watershed” and according to cerebral vascular territories in anterior circulation - media and anterior cerebral arteries, posterior circulation - vertebral arteries, basilar, and posterior cerebral. Stroke characteristics were analyzed independently and the same individual may have been classified into more than one topography, according to the lesion location. All individuals were also classified according to Bamford classification (total anterior circulation syndrome - TACS; partial anterior circulation syndrome - PACS; lacunar syndrome - LACS; posterior circulation syndrome - POCS)^15^.

#### Swallowing assessment

Swallowing was evaluated by speech language pathologists (SLP) at the bedside using the Volume-Viscosity Swallow Test (V-VST)^16^. We mixed 100 mL of water with three measures of a xanthan-based thickener to make the pudding consistency, and with one measure to make the nectar consistency. We used increasing volumes of 5-, 10-, and 20-mL boluses in a progression of increasing difficulty as proposed in the V-VST, and the presence of dysphagia was determined according to the test results. Any sign or symptom of swallowing impairment (oral residue, reduced efficiency of labial seal, fractional swallow, and pharyngeal residue) or any sign of unsafe swallowing (cough, change in voice quality and decrease in oxygen saturation ≥3%) was considered dysphagia.

#### Outcomes

Individuals were assessed for functional status during an outpatient clinic visit three months after stroke onset by raters blinded to dysphagia diagnosis during acute hospital stay. Data from individuals that died were obtained from hospital records. Functional outcomes were functional disability, assessed using the modified Rankin scale^17^ (mRS; 0-2: no functional dependence; 3-6: functional dependence or death), functional dependence, assessed using the Barthel Index^17^ and Functional Independence Measure (FIM)^18^, and use of tube feeding. Individuals were also asked if they had received any rehabilitation since their stroke onset. Individuals who could not attend their outpatient clinic appointment were contacted by phone for details to inform the mRS score, the use of tube feeding and rehabilitation.

### Data analysis

Clinical and demographic information were summarized descriptively using frequencies, percentages, means, standardized deviations (SD), medians and interquartile ranges (IQ). Data from individuals with and without dysphagia were compared using the t-test or Mann-Whitney test for continuous variables and chi-square or Fisher’s exact test for categorical variables. Multivariate logistic regression was applied using a backward stepwise method to identify the factors associated with dysphagia and to determine if dysphagia was an independent predictor of death and or of functional dependence at three months post-stroke. All variables that presented a statistically significant difference in the univariate analysis and were potential associated factors for dysphagia and for functional dependence or death were included in the multivariate logistic regression model. We used the threshold of 0.05 for statistical significance for all analyses. Statistical analyses were performed using the software SPSS version 20.

## RESULTS

**Figure 1.**
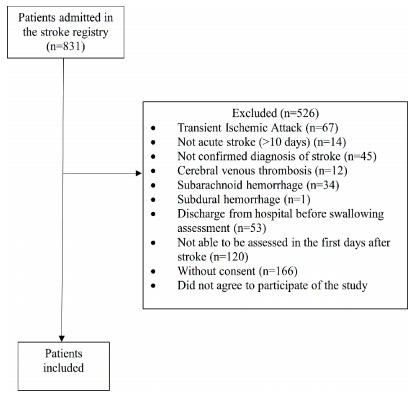
Flowchart of the study.

A total of 831 individuals with suspected stroke were admitted to hospital between April 2015 and September 2016. Of these, 305 individuals were included based on inclusion and exclusion criteria (Figure 1). The mean age of individuals was 63.6±13.3 years, 168 (55.1%) were male, 285 (93.4%) had an ischemic stroke, median National Institutes of Health Stroke Scale (NIHSS) score at admission was 7^4-13^, and 18 (5.9%) individuals died within three months after stroke. The mean time from stroke to swallowing assessment was 4.2±4.1 days.

### Factors associated with dysphagia

One hundred and thirty-eight (45.2%) individuals had dysphagia, 54.7% of which had only safety impairments, 9.4% had only efficacy impairments, and 35.9% had both safety and efficacy impairments. Cough was the most frequent sign of impaired safe swallow (47.3%) and reduced efficiency of labial seal was the most frequent sign of impaired efficacy (41.8%). Dysphagic individuals were older (65.8±13.3 vs. 61.9±13.0 years; p=0.010) and had higher stroke severity at hospital admission (9^5-17^ vs. 5^3-10^; p<0.001) than comparable individuals without dysphagia. They were also more likely to have obstructive sleep apnea (OSA) (12.3 vs. 3%; p=0.002) and TACS (18.8 vs. 9.6%; p=0.019) and less likely to have LACS (21.7% vs. 36.5%; p=0.005) than individuals without dysphagia (Table 1). In the multivariate analysis, age (OR=1.02; 95%CI 1.00-1.04; p=0.017), medical history of OSA (OR=5.13; 95%CI 1.74-15.15; p=0.003), and stroke severity at hospital admission (OR=1.10; 95%CI 1.06-1.15; p<0.001) were independently associated with dysphagia (Table 2).

**Table 1.** Baseline characteristics of patients with and without dysphagia.

	General n=305	Dysphagia		
		Yes n=138	No n=167	p-value
Age (years), mean±SD	63.6±13.3	65.8±13.3	61.9±13.0	0.010*
Male sex	168 (55.1%)	77 (55.8%)	91 (54.5%)	0.819
Pre-event functional dependence	17 (5.6%)	10 (7.2%)	7 (4.2%)	0.252
Prior stroke	91 (29.8%)	43 (31.2%)	48 (28.7%)	0.646
Hypertension	232 (76.1%)	98 (71.0%)	134 (80.2%)	0.060
Diabetes	99 (32.5%)	47 (34.1%)	52 (31.1%)	0.588
Dyslipidemia	107 (35.1%)	46 (33.3%)	61 (36.5%)	0.561
Obstructive sleep apnea	22 (7.2%)	17 (12.3%)	5 (3.0%)	0.002*
Atrial fibrillation	58 (19.0%)	28 (20.3%)	30 (18.0%)	0.606
Cardiac insufficiency	37 (12.1%)	21 (15.2%)	16 (9.6%)	0.133
Obesity	66 (22.5%)	31 (23.0%)	35 (22.2%)	0.841
Smoking in the past year	82 (30.0%)	44 (34.4%)	40 (26.3%)	0.143
Alcoholism in the past year	119 (42.8%)	50 (39.4%)	69 (45.7%)	0.288
GCS at admission, median [IQ]	15 [14-15]	15 [14-15]	15 [14-15]	0.144
NIHSS at admission, median [IQ]	7 [4-13]	9 [5-17]	5 [3-10]	<0.001*
Thrombolysis or thrombectomy	92 (30.2%)	47 (34.1%)	45 (26.9%)	0.178
Thrombolysis	82 (26.9%)	42 (51.2%)	40 (48.8%)	0.180
Thrombectomy	37 (12.1%)	20 (54.1%)	17 (45.9%)	0.290
Hemorrhagic stroke	20 (6.6%)	11 (8.0%)	9 (5.4%)	0.365
Right hemisphere	137 (52.7%)	64 (55.2%)	73 (50.7%)	0.472
Bamford TACS	42 (13.8%)	26 (18.8%)	16 (9.6%)	0.019*
Bamford PACS	131 (43.0%)	61 (44.2%)	70 (41.9%)	0.688
Bamford LACS	91 (29.8%)	30 (21.7%)	61 (36.5%)	0.005*
Bamford POCS	40 (13.1%)	20 (14.5%)	20 (12.0%)	0.517
Anterior circulation	263 (86.2%)	118 (85.5%)	145 (86.8%)	0.739
Watershed	13 (4.6%)	5 (3.9%)	8 (5.1%)	0.651
Cerebellum	18 (6.3%)	6 (4.7%)	12 (7.6%)	0.322
Cortical	125 (43.9%)	57 (44.9%)	68 (43.0%)	0.755
Lacuna	80 (26.2%)	31 (22.5%)	49 (29.3%)	0.174

*statistically significant; GCS: Glasgow Coma Scale; NIHSS: National Institutes of Health Stroke Scale*;* IQ: interquartile range; SD: standardized deviation; TACS: total anterior circulation syndrome; PACS: partial anterior circulation syndrome; LACS: lacunar syndrome; POCS: Posterior circulation syndrome.

**Table 2.** Multivariate analysis of dysphagia predictors.

		OR	95%CI	p-value
Step 1	Age	1.02	1.00-1.04	0.021
	Stroke severity	1.09	1.04-1.14	<0.001
	Obstructive sleep apnea	5.51	1.84-16.49	0.002
	TACS	1.00	0.45-2.20	0.986
	LACS	0.66	0.37-1.17	0.157
Step 2	Age	1.02	1.00-1.04	0.021
	Stroke severity	1.09	1.05-1.14	<0.001
	Obstructive sleep apnea	5.50	1.84-16.48	0.002
	LACS	0.66	0.37-1.16	0.151
Step 3	Age	1.02	1.00-1.04	0.017
	Stroke severity	1.10	1.06-1.14	<0.001
	Obstructive sleep apnea	5.13	1.73-15.14	0.003

OR: *Odds Ratio*; 95%CI: 95% confidence interval; TACS: total anterior circulation syndrome; LACS: lacunar syndrome.

### Outcomes

**Figure 2.**
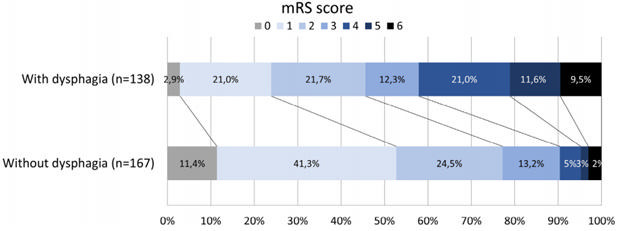
Modified Rankin Scale score (0 to 6; 0=no symptoms, 6=dead) at three months post-stroke in individuals with and without dysphagia diagnosed in the acute phase.

Dysphagic individuals had longer length of hospital stay (10.0±10.2 vs. 6.7±7.8 days; p=0.001), used tube feeding during hospitalization (65.1 vs. 18%), had functional dependence at discharge (mRS 3-6: 54.3 vs. 22.8%), did rehabilitation (58.2% vs. 31.8%), and used tube feeding (15.5 vs. 0.9%) within three months more often (p<0.001 for all comparisons) than individuals without dysphagia (Table 3). They also were more likely to die (9.4 vs. 3%; p=0.010) and were more functionally dependent (Barthel: 71.1±33.1 vs. 91.0±16.9; FIM: 92.3±35.1 vs. 114.4±19.8; p<0.001) at three months (Figure 2). Presence of dysphagia (OR=3.78; 95%CI 2.16-6.61; p<0.001) and stroke severity as measure by the NIHSS scale (OR=1.05; 95%CI 1.00-1.09; p=0.024) were independently associated with death and functional dependence at three months (Table 4).

**Table 3.** Outcomes of patients with and without dysphagia.

	General (n=305)	**Dysphagia**		
		Yes (n=138)	No (n=167)	p-value
Length of stay (days), mean±SD	8.2±9.0	10.0±10.2	6.7±7.8	0.001
Use of tube feeding in hospital	107 (40.4%)	82 (65.1%)	25 (18.0%)	<0.001
mRS score at discharge				
0-2	151 (53.0%)	41 (32.3%)	110 (69.6%)	<0.001
3-6	134 (47.0%)	86 (67.7%)	48 (30.4%)	
In-hospital death	3 (1.0%)	2 (1.4%)	1 (0.6%)	0.454
mRS score at 3 months				
0-2	192 (63.0%)	63 (45.7%)	129 (77.2%)	<0.001
3-6	113 (37.0%)	75 (54.3%)	38 (22.8%)	
Barthel at 3 months, mean±SD	82.4±27.0	71.1±33.1	91.0±16.9	<0.001
FIM at 3 months, mean±SD	104.9±29.5	92.3±35.1	114.4±19.8	<0.001
Death within 3 months	18 (5.9%)	13 (9.4%)	5 (3.0%)	0.018
Rehabilitation within 3 months	88 (43.8%)	53 (58.2%)	35 (31.8%)	<0.001
SLP therapy	22 (12.0%)	14 (18.2%)	8 (7.5%)	0.029
Physiotherapy	66 (35.9%)	35 (46.1%)	31 (28.7%)	0.016
Use of tube feeding within 3 months	14 (7.4%)	13 (15.5%)	1 (0.9%)	<0.001

SD: standard deviation; mRS: modified Rankin Scale; FIM: functional Independence Measure; SLP: speech and language pathology.

**Table 4.** Multivariate analysis for predictors of functional dependence or death at three months.

		OR	95%CI	p-value
Step 1	Age	1.01	0.99-1.04	0.181
	Stroke severity	1.04	0.99-1.09	0.157
	TACS	1.29	0.55-3.00	0.555
	LACS	0.69	0.35-1.35	0.279
	Dysphagia	3.52	2.00-6.20	<0.001
Step 2	Age	1.01	0.99-1.04	0.169
	Stroke severity	1.04	1.00-1.09	0.072
	LACS	0.67	0.35-1.31	0.243
	Dysphagia	3.53	2.00-6.20	<0.001
Step 3	Age	1.01	0.99-1.04	0.153
	Stroke severity	1.05	1.01-1.10	0.022
	Dysphagia	3.60	2.05-6.32	<0.001
Step 4	Stroke severity	1.05	1.01-1.09	0.024
	Dysphagia	3.78	2.16-6.61	<0.001

TACS: total anterior circulation syndrome; LACS: lacunar syndrome.

## DISCUSSION

The frequency of dysphagia identified in our study is consistent with two recent cohort studies^14^ ^,^ ^15^, one of them in Brazil^14^. However, it was lower than the frequency of dysphagia estimated in a systematic review of Brazil [13], probably because we did not include severely affected individuals who could not be evaluated in the first days after the stroke.

The results of our study confirm the association of dysphagia with age and stroke severity reported in the literature^5^ ^,^ ^11^ ^,^ ^14^ ^,^ ^19^ ^,^ ^20^ ^,^ ^21^. Thus, among the many factors associated with dysphagia in stroke individuals, age and stroke severity are strong risk factors for dysphagia. Therefore, older individuals and those with more severe strokes should be monitored more carefully due to the risk of developing dysphagia and its associated complications.

An important finding in our study was the association between OSA and dysphagia. OSA is an important risk factor for stroke^22^ ^,^ ^23^ ^,^ ^24^, being common in this population. It is also associated with changes in swallowing^25^ ^,^ ^26^ ^,^ ^27^ ^,^ ^28^. However, to date, no study has investigated whether individuals with stroke and OSA are at greater risk of developing dysphagia than individuals with stroke without OSA. In our study, OSA was an independent risk factor for dysphagia, so these individuals are more likely to develop dysphagia after a stroke. Thus, all stroke patients with OSA should be assessed for orofacial muscles and swallowing, as they have more chances to have dysphagia compared to stroke individuals without OSA.

Our study also confirmed that dysphagia in stroke patients in Brazil has an important impact on length of stay, mortality, and outcomes^9^ ^,^ ^14^. This highlights the importance of promoting adequate management strategies for dysphagia in Brazilian guidelines to avoid poor outcomes in this population.

Thus, this study provides important epidemiological data for stroke care in Brazil to help identify individuals at risk for dysphagia and to demonstrate the impact of dysphagia on this population. This highlights the importance of promoting better management strategies for these individuals to prevent poor outcomes. These strategies include screening for early detection of dysphagia and referring individuals who do not have it for evaluation by speech therapists.

There are some limitations in this study. Data about risk factors for stroke were collected from the medical history reported by the individual or his or hers caregiver. However, the presence of risk factors such as OSA was considered only if the diagnosis was confirmed or if the person was taking medication for the condition. We did not evaluate more severe stroke individuals because we only assessed individuals in the first few days after hospital admission, and severe patients could not be assessed at that time. Despite this, we could identify a high rate of dysphagia and associations with poor outcomes.

Despite these limitations, our study was performed prospectively and consecutively, with a large cohort of stroke individuals, and all individuals were assessed in a standardized way by SLP. The outcomes were assessed blindly for dysphagia diagnosis, which contributes to the reliability of the observed results.

This study confirms that dysphagia is frequent in post-stroke individuals and is a strong predictor of death or functional dependence. Stroke teams should be alert for increased risk of dysphagia in the elderly or those with OSA. Brazilian health managers should be aware of the need to implement strategies for early detection and management of dysphagia to avoid poor outcomes.
